# *ACTN3* Variants Beyond Performance: Metabolic and Inflammatory Implications

**DOI:** 10.3390/genes17101280

**Published:** 2026-10-14

**Authors:** Antonio Imperatore, Fernanda Iafusco, Mariella Calvanese, Alessandro Gentile, Raffaele Amitrano, Fabiana Di Fusco, Marta Mallardo, Gaetano Iaquinto, Mattia Digno, Salvatore Tamburro, Ersilia Nigro, Roberto Berni Canani, Annamaria Mancini, Pasqualina Buono, Cristina Mazzaccara, Barbara Lombardo, Daniela Terracciano, Giulia Frisso, Aurora Daniele, Paola Borrelli, Cristina Mennitti, Nadia Tinto, Olga Scudiero

**Affiliations:** 1 Department of Molecular Medicine and Medical Biotechnology, University of Naples Federico II, 80131 Naples, Italy; impeantonio@gmail.com (A.I.); mariellacalvanese99@gmail.com (M.C.); alessandro.gentile@cuanschutz.edu (A.G.); raff.amitrano@studenti.unina.it (R.A.); fabiana.difusco@gmail.com (F.D.F.); cristina.mazzaccara@unina.it (C.M.); barbara.lombardo@unina.it (B.L.); giulia.frisso@unina.it (G.F.); aurora.daniele@unina.it (A.D.); olga.scudiero@unina.it (O.S.); 2 CEINGE-Biotecnologie Avanzate, via Gaetano Salvatore 486, 80045 Naples, Italy; iafusco@ceinge.unina.it (F.I.); marta.mallardo@unipegaso.it (M.M.); ersilia.nigro@unicampania.it (E.N.); berni@unina.it (R.B.C.); annamaria.mancini@uniparthenope.it (A.M.); pasqualina.buono@uniparthenope.it (P.B.); 3 NutriSportHealthLab, University of Naples Federico II, Via Sergio Pansini 5, 80131 Naples, Italy; 4 Division of Cardiology, University of Colorado School of Medicine, 12505 E. 16th Avenue, Anschutz Inpatient Pavilion 2, Aurora, CO 80045, USA; 5 Department of Education and Sports Sciences, Pegaso Telematic University, 80143 Naples, Italy; 6 SS Napoli Basket SRL, 80127 Naples, Italy; gaetanoiaquinto@gmail.com (G.I.); mattiadigno@gmail.com (M.D.); 7 Centro Universitario Sportivo di Napoli, Via Campegna 267, 80124 Naples, Italy; salvatore65tamburro@gmail.com; 8 Department of Environmental, Biological and Pharmaceutical Sciences and Technologies, University of Campania “Luigi Vanvitelli”, Via Vivaldi 43, 81100 Caserta, Italy; 9 Department of Translational Medical Sciences, University of Naples “Federico II”, 80131 Naples, Italy; daniela.terracciano@unina.it; 10 Department of Medical, Human Movement and Well-Being Sciences, University Parthenope, Via Medina 40, 80133 Naples, Italy; 11 Department of Medical, Oral and Biotechnological Sciences, Laboratory of Biostatistics, University G. d’Annunzio of Chieti-Pescara, 66100 Chieti, Italy; paola.borrelli@unich.it

**Keywords:** *ACTN3*, genetic predisposition, biochemical parameters

## Abstract

**Background:** Athletic performance is a result of interaction between genetic and environmental factors, including training, nutrition, rest, hormonal and psychophysiological status. Numerous genes and polymorphisms can influence key physiological processes, such as muscle contractility, energy metabolism, oxidative capacity, and cardiovascular efficiency. Among these, the *ACTN3* gene, and in particular the polymorphism rs1815739 (NM_001104.4:c.1729C>T; p.Arg577Ter), is well known for its association with athletic performance, but emerging evidence suggests broader effects on metabolic and inflammatory phenotypes. **Methods:** For this study, 109 elite athletes were enrolled and genotyped for *ACTN3* c.1729C>T by Sanger sequencing. Moreover, biochemical markers, such as creatine kinase (CK), C-reactive protein (CRP), lipid profile, adiponectin, and neutrophil-to-lymphocyte ratio (NLR), were examined. Finally, to better investigate the association between genotype and athletic performance, the genotypes and the results of standardized physical tests were correlated in a small exploratory subgroup of 28 professional basketball athletes. Normality was assessed using the Shapiro–Wilk test; non-normally distributed variables were summarized as median [IQR] and analyzed with non-parametric methods. Genotype comparisons were adjusted for multiple testing using the Holm procedure, and principal biochemical models were adjusted for age, BMI, sex, and ancestry. **Results:** The c.1729C>T genotype distribution differed between the two ancestry groups (exact *p* = 0.049), and all three variants were compatible with Hardy–Weinberg equilibrium within the ancestry groups. No biochemical genotype association remained statistically significant after Holm correction. In the performance subgroup, no genotype–performance association remained significant after Holm correction. **Conclusions:** In this cohort, the corrected analyses did not provide robust evidence for genotype-dependent biochemical or performance effects. The results are exploratory and require replication in larger cohorts, considering ancestry and gender differences.

## 1. Introduction

Athletic performance is a complex, multifactorial trait resulting from the interaction between genetic and environmental factors, including training, nutrition, rest, hormonal and psychophysiological status [1,2]. Advances in genomics and next-generation genotyping have identified numerous genes and polymorphisms influencing key physiological processes, such as muscle contractility, energy metabolism, oxidative capacity, and cardiovascular efficiency [3,4].

Performance is largely polygenic: multiple variants, each with modest effects, collectively contribute to an individual’s athletic potential [5]. Genome-wide association studies (GWAS) and meta-analyses have identified over 200 polymorphisms associated with endurance, power, or strength phenotypes [6]. While genetic predisposition plays a significant role, environmental factors and physical activity modulate gene expression, including through epigenetic mechanisms, emphasizing the interplay between genes and lifestyle in shaping performance [7,8]. In this context, profiling multiple genetic markers can help to describe individual predispositions toward specific performance traits and physiological adaptations, providing a basis for personalized training strategies and targeted interventions [9].

Within sports genetics, the *ACTN3* gene plays a key role due to its well-established influence on power and endurance capacities. Located on the long arm of chromosome 11, *ACTN3* encodes α-actinin-3, a structural protein localized in the Z-discs of type II fast-twitch muscle fibers, which are responsible for forceful and explosive movements. Because of its specific muscular function, the *ACTN3* gene, and, in particular, its variant rs1815739 [NM_001104.4:c.1729C>T (p.Arg577Ter) at genomic position chr11:66560624 (GRCh38)], are considered key elements in determining sprint and power performance [10,11]. The rs1815739 variant is a nonsense single nucleotide polymorphism (SNP) resulting in a C>T substitution at nucleotide position 1729. This substitution converts an arginine (Arg) codon into a premature stop codon (Ter), leading to translational arrest. Consequentially, individuals with the CC (homozygous wild-type) or CT (heterozygous) genotypes produce functional α-actinin-3, whereas TT homozygotes exhibit a complete deficiency of the protein in skeletal muscle [12]. Nevertheless, α-actinin-3 deficiency does not result in evident muscle dysfunction, as it is compensated by the upregulation of α-actinin-2, a functionally similar protein encoded by *ACTN2* [13]. Consequently, while the total sarcomeric α-actinin content remains unaltered, the absence of α-actinin-3 modifies both Z-disc protein–protein interactions and the elastic properties of fast-twitch muscle fibers. This structural remodeling provides a mechanistic basis for the reduced force generation capacity and the increased susceptibility to muscle damage during eccentric exercise observed in α-actinin-3-deficient individuals [14].

**Figure 1:**
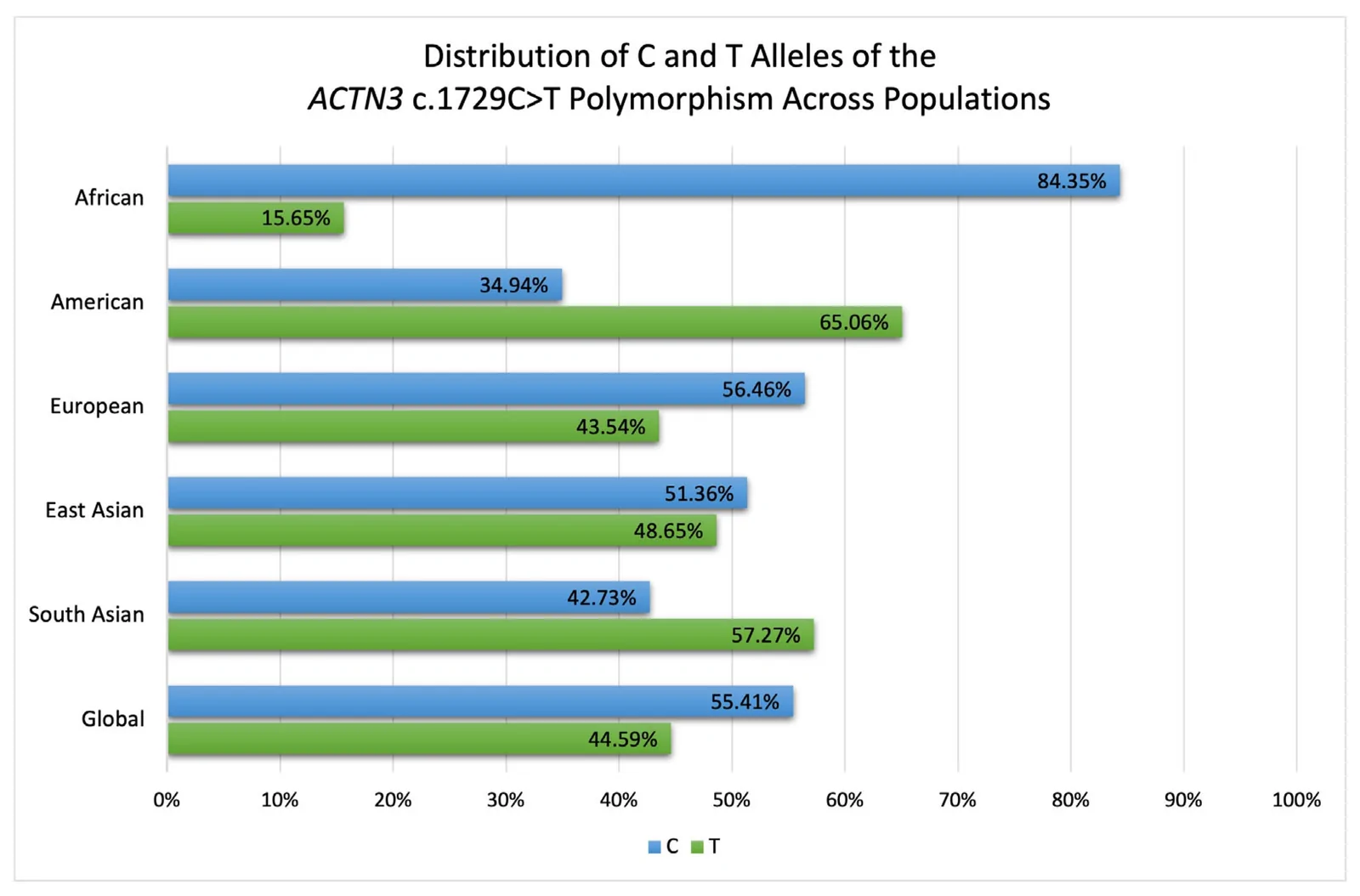
Distribution of C and T allele frequencies of the *ACTN3* c.1729C>T polymorphism in populations. Bars indicate the percentage frequencies of the two alleles in African, American, European, East Asian, South Asian, and global populations. Data were derived from gnomAD v.4 (Genome Aggregation Database) and represent allele frequencies observed in the included individuals following standard gnomAD quality control procedures [15].

Global allele frequencies of the *ACTN3* c.1729C>T polymorphism differ in populations, with a higher prevalence of the T allele in South Asia, East Asia, and the Americas compared to Africa and Europe [15] (Figure 1).

Numerous studies have investigated the functional relevance of c.1729C>T polymorphism in athletic performance, but available findings remain divergent [16,17,18]. On the other hand, evidence from recent meta-analyses have suggested a differential distribution of *ACTN3* genotypes among power athletes, with over-representation of the CC genotype and under-representation of the TT genotype, supporting the hypothesis that the C allele confers an advantage in explosive disciplines [19]. Conversely, studies in elite endurance athletes have shown a higher frequency of the T allele and the TT genotype, particularly among world-class performers, suggesting a favorable effect of the T allele on aerobic capacity [20].

Although α-actinin-3 deficiency is not associated with pathology and may even confer advantages in endurance-based sports, the TT genotype has been linked to increased susceptibility to exercise-induced muscle damage and longer recovery periods [21,22]. Mechanistically, this vulnerability is thought to arise from reduced Z-disc proteins stability under mechanical stress. Conversely the expression of α-actinin-3 enhances fiber rigidity of type IIa fibers, providing a protective effect against contraction-induced strain [23].

Beyond these acute mechanical effects, numerous evidence indicates that the *ACTN3* c.1729C>T polymorphism functions as important modifier of the muscle phenotype, influencing musculoskeletal aging, disease progression, and metabolic health throughout the lifespan.

In older adults, *ACTN3* genotype has been associated with variability in sarcopenia and osteoporotic status, with the T allele conferring increased susceptibility to loss of lean body mass (LBM) and bone mineral density (BMD). This vulnerability appears to be partially modulated by lifestyle-related factors such as body composition, physical activity, nutrition, and serum vitamin D levels, highlighting a gene–environment interaction in musculoskeletal aging. In contrast, the protective effect of the C allele against age-related muscle atrophy has been linked to its association with greater muscle mass and a higher proportion of fast-twitch fibers [24].

In the context of Duchenne Muscular Dystrophy (DMD), the *ACTN3* genotype acts as a subtle genetic modifier of disease progression, influencing both skeletal muscle characteristics and cardiac outcomes. Patients with the TT genotype of c.1729C>T polymorphism, who lack α-actinin-3, tend to exhibit a shift toward a slower, more oxidative muscle phenotype, whereas carriers of the C allele (CC or CT) show a higher proportion of fast-twitch fibers and greater baseline muscle strength [25]. Beyond skeletal muscle, emerging clinical evidence indicates that the *ACTN3* 577TT genotype is also associated with earlier and more severe cardiac involvement, particularly an increased risk of left ventricular dilation and cardiomyopathy [26].

In addition, c.1729C>T polymorphism has also been associated with metabolic and cardiovascular markers. In rugby players, the TT genotype correlated with higher HDL cholesterol and adiponectin, suggesting a potential cardioprotective effect [27]. However, other studies report that TT individuals may exhibit higher glucose, triglycerides, and total cholesterol, with lower HDL levels, and although TT is more common in type 2 diabetes, it does not appear to significantly affect glycemic control or obesity [28,29].

Taken together, these findings highlight that the *ACTN3* c.1729C>T polymorphism has implications that extend beyond athletic performance, influencing musculoskeletal integrity, metabolic health, and disease susceptibility across physiological and pathological conditions. This growing body of evidence highlights the clinical relevance of *ACTN3* genotyping and supports the need for integrated approaches that consider both performance-related and health-related outcomes.

While genetic polymorphisms such as *ACTN3* c.1729C>T provide insight into an individual’s predisposition to specific performance traits, biochemical parameters reflect the acute and chronic responses to training [30]. Therefore, the integration of laboratory medicine with molecular genetics offers a more comprehensive approach to athlete evaluation, supporting personalized training strategies, performance optimization, and injury prevention.

In this context, the present study aims to evaluate: (i) the role of the *ACTN3* c.1729C>T polymorphism (rs1815739), including (ii) creatine kinase (CK), lactate dehydrogenase (LDH), alkaline phosphatase (ALP), C-reactive protein (CRP), lipid profile, and adiponectin, in a group of elite athletes, providing insights relevant not only for sports science but also for clinical applications.

## 2. Materials and Methods

### 2.1. Ethical Approval

The study was conducted in accordance with the ethical guidelines of Helsinki Declaration of the World Medical Association and was approved by Campania Territorial Ethics Committee 3 (protocol 264/2024).

### 2.2. Participants

For this study, between 2018 and 2025, a total of 109 elite athletes competing in different sports disciplines were recruited. All participants were officially registered athletes and were regularly monitored by the technical and medical staff of their respective sports clubs throughout the competitive seasons. The study population included 7 female athletes practicing synchronized swimming and 102 male athletes, of whom 90 were basketball players and 12 were water polo players. The cohort was ethnically heterogeneous, comprising 75 Caucasian athletes and 34 athletes of African descent. The physical characteristics of the players as mean (±SD) were age 24.5 ± 6.3 years, weight 88.5 ± 14.1 kg, height 1.9 ± 0.1 m and BMI 23.7 ± 1.9. No specific inclusion or exclusion criteria were applied; therefore, the entire roster of athletes officially enrolled during the observation period was analyzed, ensuring representativeness of the reference athletic population. Physical performance test results from a subgroup of 28 basketball players were obtained from reports provided by the same strength and conditioning coach, who supervised the athletes over the last three competitive seasons. The available dataset included one performance record per athlete; therefore, repeated observations from the same athlete across different seasons were not available for the present analysis. Written informed consent was obtained from all participants prior to blood sampling. Participants were informed about the aims of the study, the biological samples to be collected, the analytical procedures, and the protocols to be followed.

### 2.3. Biochemical Analyses

Blood samples were collected at approximately 8:00 a.m. after an overnight fast and following at least 72 h of rest from physical exercise or competition. Biochemical parameters indicative of inflammatory status like creatine kinase (CK), lactate dehydrogenase (LDH), alkaline phosphatase (ALP), and C-reactive protein (CRP), together with lipid profile parameters (total cholesterol, LDL cholesterol, HDL cholesterol, and triglycerides), were measured in serum samples at the Clinical Biochemistry Unit using an ARCHITECT c16000 automated analyzer (Abbott Laboratories, Chicago, IL, USA) based on spectrophotometric methods. The total serum adiponectin concentration was determined using an enzyme-linked immunosorbent assay (ELISA) with a polyclonal antibody developed in-house against a human adiponectin amino acid fragment (H2N-ETTTQGPGVLLPLPKG-COOH), as previously described [31]. Each sample was analyzed in duplicate, with two independent measurements performed for accuracy. Complete blood counts were performed on EDTA-anticoagulated whole blood at the Laboratory Hematology Unit using a Siemens Advia 2120i hematology analyzer (Tarrytown, USA). Absolute neutrophil and lymphocyte counts were used to calculate the neutrophil-to-lymphocyte ratio (NLR) according to the standard formula, which was considered an index of systemic inflammation reflecting the balance between innate (neutrophils) and adaptive (lymphocytes) immune responses.

### 2.4. Genetic Analysis

Genomic DNA was extracted from leukocytes obtained from peripheral blood samples collected in EDTA-treated tubes using the QIAamp DNA Mini Kit (Qiagen, Hilden Germany), following the manufacturer’s standard protocol. DNA concentration and purity were assessed by UV–visible spectrophotometry using a NanoDrop™ 2000 instrument (Thermo Scientific, Wilmington, Delaware USA). Molecular analysis of the *ACTN3* gene was performed by polymerase chain reaction (PCR) amplification of all coding exons and flanking intronic regions using gene-specific primers designed based on the reference sequence (NCBI accession number NM_001104.4): forward primer 5′-CTGTTGCCTGTGGTAAGTGGG-3′ and reverse primer 5′-TGGTCACAGTATGCAGGAGGG-3′. PCRs were carried out according to standard protocols using a commercial PCR Master Mix (Promega Corporation, Fitchburg, WI, USA) and included negative controls to exclude contamination. Amplification products were verified by agarose gel electrophoresis. Bidirectional DNA sequencing was performed using the Sanger method with the BigDye Terminator Cycle Sequencing Kit (Life Technologies, Vilnius Lithuania) and analyzed by capillary electrophoresis on an ABI Prism 3730 Genetic Analyzer (Applied Biosystems, Foster City, CA, USA). Obtained sequences were aligned and compared with reference sequences available in the GenBank database using CodonCode Aligner software version 12.

### 2.5. Statistical Analysis

Descriptive statistics were calculated for all variables. Distributional normality of continuous variables was assessed using the Shapiro–Wilk test, together with graphical inspection. A two-sided Shapiro–Wilk *p*-value > 0.05 was considered compatible with the assumption of normality. Normally distributed variables were summarized as mean ± standard deviation (SD), whereas non-normally distributed variables were summarized as median and interquartile range (IQR). Categorical variables were reported as absolute frequencies and percentages. For genotype-group comparisons, one-way ANOVA was used for approximately normally distributed outcomes when assumptions were adequate; otherwise, the Kruskal–Wallis test was used. Exact tests were used for categorical comparisons when expected cell counts were insufficient for asymptotic chi-square testing. Hardy–Weinberg equilibrium was assessed separately within the Caucasian and Afri-can-ancestry groups using an exact test. Principal biochemical models included genotype as a categorical predictor and were adjusted for age, BMI, sex, and ancestry; robust standard errors were used. Effect sizes with 95% confidence intervals (CIs) were reported. For biochemical analyses, the multiple-testing family comprised the 10 prespecified biochemical/inflammatory/lipid outcomes evaluated for each ACTN3 variant; for performance analyses, the family comprised the 7 prespecified performance outcomes evaluated for each variant. Both unadjusted and Holm-adjusted *p*-values were reported. A two-sided *p*-value < 0.05 was considered statistically significant before adjustment, and a Holm-adjusted *p*-value < 0.05 was considered statistically significant after multiplicity correction. All statistical analyses were performed using Stata version 18.0 MP (StataCorp, College Station, TX, USA).

## 3. Results

### 3.1. Genotyping of the ACTN3 c.1729C>T (rs1815739) Polymorphism and Identification of Additional Variants

In line with the objectives of the project, 109 elite athletes from different sporting disciplines and sex were analyzed by Sanger sequencing for the *ACTN3* c.1729C>T (rs1815739) polymorphism in order to describe the distribution of the CC, CT, and TT genotypes within the study cohort (Table 1).

The estimated allele frequencies were 69.72% for the C allele and 30.28% for the T allele, values comparable to those reported in the gnomAD database. Because the study did not include a matched local non-athlete control group, these frequencies were not interpreted as evidence of athlete-specific selection.

Although the study was primarily aimed at genotyping athletes for the *ACTN3* c.1729C>T polymorphism, analysis of the sequencing data unexpectedly allowed the identification of two additional polymorphisms: rs2229455 [NM_001104.4:c.1689A>G (p.Thr563=) at genomic position chr11:66560584 (GRCh38)], characterized by an A>G nucleotide substitution at position 1689, which at the protein level corresponds to a synonymous substitution of threonine (Thr563=); and a second variant, NM_001104.4:c.1693 C>G, (p.His565Asp) at genomic position chr11:66560588 (GRCh38), characterized by a C>G nucleotide substitution at position 1693, resulting in the substitution of histidine with aspartic acid at the protein level (His565Asp).

Systematic searches across public repositories, including gnomAD (v4.0), ClinVar, dbSNP, Ensembl, and PubMed, yielded no prior reports or assigned rsIDs for the c.1693C>G (p.His565Asp) substitution, confirming that it represents a novel variant.

The study population was also stratified according to the genotypes of these two variants.

The results concerning the distribution of genotypes for the *ACTN3* Thr563= (rs2229455) polymorphism in the study population are reported in Table 2.

The estimated allele frequencies were 81.7% for the A allele and 18.3% for the G allele, values comparable to those reported in the gnomAD database. These frequencies are reported descriptively and are not interpreted as evidence of athlete-specific enrichment.

The results concerning the distribution of genotypes for the *ACTN3* His565Asp polymorphism in the study population are reported in Table 3.

The estimated allele frequencies were 93.6% for the C allele and 6.4% for the G allele. At present, no reference data is available in genetic databases; therefore, no comparison is possible. Moreover, the absence of homozygous individuals for this variant should be considered.

The distribution of genotypes for the three ACTN3 variants was further evaluated according to the two main ancestral groups represented in the study population: individuals of Caucasian (*n* = 75) and African ancestry (*n* = 34) (Table 4).

For the *ACTN3* c.1729C>T polymorphism (rs1815739), a statistically significant difference in overall genotype distribution was observed between the two ancestral groups (exact *p* = 0.049). In particular, individuals of African ancestry showed a higher proportion of the CC genotype, whereas Caucasian individuals more frequently carried the CT genotype. The TT genotype was infrequent in both populations. These findings are consistent with allele and genotype frequency distributions reported in large population databases, such as gnomAD (Figure 1).

Conversely, the c.1689A>G and c.1693C>G distributions did not differ significantly between ancestry groups (exact *p* = 0.525 and *p* = 0.542, respectively). HWE exact-test *p*-values were 0.348 and 0.052 for c.1729C>T in the Caucasian and African-ancestry groups, respectively; 0.444 and 0.336 for c.1689A>G; and 1.000 and 1.000 for c.1693C>G. These analyses did not show statistically significant departure from HWE at *p* < 0.05.

Because ancestry was associated with c.1729C>T genotype distribution, ancestry was retained as a covariate in the multivariable biochemical models. The observed ancestry difference should not be interpreted as evidence that a genotype is specific to elite athletes in the absence of a matched non-athlete control group.

### 3.2. Association Between Genotype and Biochemical/Inflammatory Parameters and Lipid Profile

To evaluate potential associations between genotypes for the three *ACTN3* polymorphisms and biochemical, inflammatory, and lipid profile parameters, serum levels of creatine kinase (CK), lactate dehydrogenase (LDH), alkaline phosphatase (ALP), C-reactive protein (CRP), total cholesterol, LDL cholesterol, HDL cholesterol, triglycerides and adiponectin were measured. In addition, the neutrophil-to-lymphocyte ratio (NLR) was calculated from absolute neutrophil and lymphocyte counts obtained from the complete blood count, as an additional indicator of inflammatory status.

For the *ACTN3* c.1729C>T (rs1815739) polymorphism, unadjusted analyses revealed a borderline association for lower adiponectin levels (*p* = 0.050) in individuals homozygous for the variant allele T (Table 5). In addition, this group showed non-significant trends toward higher LDL and lower HDL concentrations. A trend toward higher CK values was also observed in the CC group, consistent with the known role of the c.1729C>T polymorphism in modulating muscle fiber composition and susceptibility to muscle damage. No additional genotype-dependent differences were observed for the other biochemical or inflammatory markers. However, none remained statistically significant after Holm correction within the corresponding variant-specific testing family. Multivariable models adjusted for age, BMI, sex, and ancestry likewise did not show genotype associations that remained statistically significant after Holm correction. Results are therefore interpreted as exploratory associations rather than causal effects.

The same analyses were performed for genotypes of the *ACTN3* **c.1689A>G** (rs2229455) polymorphism, yielding the following results (Table 6). For c.1689A>G (rs2229455), unadjusted *p*-values were 0.044 for total cholesterol, 0.026 for HDL cholesterol, and 0.015 for adiponectin when the appropriate distribution-based tests were used. None remained statistically significant after Holm correction. The small GG group (*n* = 5) limits precision.

For *ACTN3* c.1693C>G (p. His565Asp), no biochemical comparison remained statistically significant after Holm correction. The NLR comparison had an unadjusted *p* = 0.066, and the absence of the GG genotype precluded estimation of a homozygous variant effect (Table 7).

### 3.3. Genotype–Physical Performance Correlations

In a subgroup of 28 professional basketball players, physical performance data were analyzed to explore potential associations between genotype and athletic performance. The assessed parameters included tests of explosive power, comprising vertical jump performance (Squat Jump, Countermovement Jump, and Drop Jump) and lateral power (right and left Lateral Bound), as well as linear speed (10m sprint) and change-of-direction ability (5-0-5 agility test). For each analyzed polymorphism, the results of these tests were correlated with genotype to evaluate potential performance differences attributable to genetic variability. Because genotype groups were small and unbalanced, distribution-free methods were used for genotype comparisons. Multiple testing across the seven performance outcomes was addressed using the Holm procedure. The available dataset did not provide sufficient detail to independently verify whether all athletes underwent identical testing sessions, equipment, timing, and assessor procedures; this limitation is therefore acknowledged.

No genotype–performance association of the three polymorphisms in the *ACTN3* gene (c.1729C>T (rs1815739), c.1689A>G (rs2229455), and c.1693C>G (p.His565Asp)) in relation to performance outcomes across the physical tests in the examined sample (*n* = 28) remained statistically significant after Holm correction. The performance analysis was considered exploratory because of the small and unbalanced genotype groups. For the *ACTN3* c.1729C>T (rs1815739) polymorphism, a slight trend toward faster 10-m sprint times was observed in individuals with the CC genotype, in agreement with the existing literature associating the C allele with enhanced muscular power. However, after applying the Holm correction for multiple comparisons, no statistically significant associations were detected across any of the evaluated athletic performance variables. Unadjusted analyses revealed borderline tendencies for 10 m speed (*p* = 0.050) and right Lateral Bound distance (*p* = 0.053). Overall, the low overall sample size (*n* = 28) and the small number of TT homozygotes (*n* = 3) limit statistical power, precluding firm genotype-specific inferences (Table 8).

Regarding the *ACTN3* c.1689A>G (rs2229455) polymorphism, no statistically significant associations were identified across any athletic performance metrics following Holm adjustment. Unadjusted analyses showed a weak, non-significant trend for 10 m sprint times, with G allele carriers exhibiting slightly faster mean times compared to AA homozygotes. However, the severe imbalance in genotype distribution substantially reduces statistical power and precludes reliable genotype–phenotype inference for this variant (Table 9).

For the *ACTN3* c.1693C>G (p.His565Asp) polymorphism, no differences among genotypes were observed across the physical performance tests considered. It should be noted, however, that no individuals homozygous for the variant allele were identified in the analyzed cohort; therefore, any potential genotype-specific effect cannot be adequately assessed. Moreover, this variant is not reported or characterized in the available literature, preventing comparison with previously published data (Table 10).

## 4. Discussion

The expanding field of sports genomics offers promising opportunities for both optimizing athletic performance and personalizing exercise-based interventions [32]. Polymorphisms in various genes can influence physical and athletic performance as well as susceptibility to injuries and metabolic disorders, providing insights for personalized training, rehabilitation, and chronic disease prevention strategies [33]. This preliminary, cross-sectional study aimed to investigate the role of *ACTN3* polymorphisms, focusing on c.1729C>T (rs1815739), by assessing potential correlations with biochemical parameters to evaluate inflammatory status, susceptibility to muscle damage, and, through lipid profiling, potential links to metabolic risk.

In an initial analysis, our data confirmed a statistically significant difference in the frequency of the *ACTN3* c.1729C>T (rs1815739) polymorphism between Caucasian and African-ancestry populations (exact *p* = 0.049). This finding is consistent with observations reported in major genomic databases such as GnomAD [15] (Figure 1) and does not demonstrate athlete-specific selection because no matched local non-athlete control group was included. Ancestry was therefore considered a potential confounder in the multivariable analyses. Specifically, the wild-type (C) allele was significantly more frequent in individuals of African ancestry compared to Caucasians, suggesting a potential advantage in power-related performance for this population.

Although no associations retained statistical significance following adjustment for multiple testing, our exploratory analysis highlights a potential metabolic pattern. Specifically, individuals homozygous for the variant allele (TT) exhibited lower adiponectin levels in the unadjusted analysis (*p* = 0.050), alongside numerically higher LDL and lower HDL concentrations. Collectively, these data may suggest differences in selected metabolic parameters according to ACTN3 genotype; however, they do not provide sufficient evidence to conclude that α-actinin-3 deficiency is associated with a less favorable cardiometabolic profile. These results contrast with those reported by Nirengi et al. [27], in which individuals with the TT-genotype exhibited higher adiponectin and HDL levels, suggesting a potential protective role against metabolic risk. However, this discrepancy may reflect differences in the study populations. While Nirengi et al. focused on elite Asian athletes, a group characterized by extreme physical conditioning and a high ancestral frequency of the T allele (Figure 1), our study involves a more diverse cohort of elite Caucasian and African-ancestry athletes. Ethnic background and lifestyle-related factors may modulate the phenotypic impact of ACTN3, and these differences between study populations could contribute to the divergent metabolic patterns observed. Given that the adiponectin association did not remain significant after Holm correction and that the LDL and HDL differences were not statistically significant in the unadjusted analyses, these findings should be considered exploratory and hypothesis-generating. We observed a descriptive trend toward higher CK levels among C-allele carriers, particularly individuals with the CC genotype, compared with TT individuals. This elevation may reflect a physiological response to intense physical activity and could be related to the muscular characteristics associated with *ACTN3* genotype. The presence of α-actinin-3 in CC individuals facilitates more efficient activation of type II muscle fibers, faster muscle metabolism, and enhanced strength and power performance [16]. Moreover, α-actinin-3 contributes to the stabilization of contractile structures, potentially reducing the risk of substantial injury despite increased acute mechanical stress. However, the present data does not allow us to establish whether the observed CK pattern reflects differences in muscle damage or exercise-related adaptation. In contrast, TT individuals, who lack α-actinin-3, showed numerically lower CK levels in the present cohort, suggesting a reduced inflammatory response. However, lower contractile efficiency may result in the gradual accumulation of micro-damage, prolonged recovery periods, and greater post-exercise discomfort [21]. Importantly, this difference was not statistically significant; therefore, the observed CK pattern should be interpreted cautiously and considered exploratory rather than evidence of a confirmed genotype-dependent difference in muscle damage or inflammatory response.

When examining standardized physical tests in a subgroup of athletes, these genotype-dependent effects were less pronounced. No clear differences were observed overall, although CC individuals displayed a slight advantage in the 10 m sprint, consistent with the literature linking the C allele to enhanced explosive power [12]. Other deviations, such as those observed in the Lateral Bound test, are more likely attributable to the relatively small subgroup sizes and uneven genotype distribution rather than providing evidence of a true biological effect. Overall, the performance findings provide limited evidence of a consistent ACTN3 genotype-dependent advantage and should therefore be interpreted as exploratory.

Sequencing analysis identified two unexpected polymorphisms, including *ACTN3* c.1689A>G (rs2229455), which yielded notable observations. This synonymous variant is relatively rare in the general population (global frequency ~17.5% according to gnomAD). Although it does not alter the amino acid sequence, our preliminary data suggest it may have subtle regulatory effects on gene expression, potentially influencing enzymatic levels and downstream physiological processes.

In our cohort, no clear genotype-dependent differences were observed for markers of muscle damage or systemic inflammation. CK, LDH, and ALP levels were broadly comparable across genotype groups, although CK and ALP showed some numerical variation, particularly in the small GG group. Similarly, CRP and NLR did not differ meaningfully between genotypes, providing no evidence of a genotype-dependent alteration in systemic inflammatory status. These findings suggest that, within the present cohort, the c.1689A>G polymorphism is not associated with consistent differences in the biochemical markers of muscle damage or inflammation examined.

More notable differences emerged for some lipid and metabolic parameters. In the initial analyses, total cholesterol, HDL cholesterol, and adiponectin differed across genotype groups, with GG individuals showing the lowest values for all three biomarkers. However, these associations did not remain statistically significant after Holm correction for multiple comparisons, and the multivariable analyses similarly did not provide robust evidence of genotype-dependent effects. Nevertheless, the consistency in the direction of these three metabolic markers may warrant further investigation, particularly because the observed effect sizes were not negligible. At the same time, the very small number of GG individuals (*n* = 5) substantially limits the precision of these estimates and makes it difficult to distinguish a potential biological signal from random variation.

These preliminary observations raise the possibility that the synonymous Thr563= variant may be associated with subtle differences in metabolic phenotype, although the present data do not establish such an association. Importantly, the absence of a significant finding after multiple-testing correction argues against interpreting the observed differences as evidence of a confirmed cardiometabolic effect. Whether the pattern observed for total cholesterol, HDL cholesterol, and adiponectin reflects a biological consequence of the variant or simply sampling variability remains to be determined. To our knowledge, this is the first study addressingc.1689A>G (rs2229455), as most of the literature has focused on the well-characterized c.1729C>T (rs1815739) variant. Given the observational design and limited sample size, caution is warranted; however, these preliminary results highlight the need for further functional studies to elucidate the potential role of c.1689A>G (rs2229455) in modulating inflammatory and metabolic biomarkers. In addition to c.1689A>G (rs2229455), sequencing revealed another novel variant, *ACTN3* c.1693C>G (p.His565Asp), which, to our knowledge, has not been previously reported in the literature or included in genetic databases. Given its missense nature, this variant may have structural and functional implications on α-actinin-3, potentially affecting protein stability, contractile function, or enzymatic interactions. Further studies, including in silico modeling and functional assays, are warranted to elucidate the potential impact of the c.1693C>G (p.His565Asp) variant on muscle physiology and related metabolic pathways. Therefore, the present study highlights the potential role of genetic variability in shaping human physiology, including physical performance, susceptibility to musculoskeletal injuries, and metabolic traits. Among well-characterized variants, the *ACTN3* c.1729C>T (rs1815739) polymorphism is known to influence explosive power and sprint performance, but these effects were not clearly evident in our cohort, possibly reflecting the limited sample size, unequal genotype distribution, and population heterogeneity.

However, our findings draw attention to the synonymous *ACTN3* c.1689A>G (rs2229455) variant, which, despite not altering the amino acid sequence, may warrant further investigation for potential regulatory effects. Such variants are often overlooked in sports genomics, yet our preliminary data provides a basis for further investigation of their potential effects on metabolic parameters, warranting further functional investigation. Of particular note is the missense *ACTN3* c.1693C>G (p.His565Asp) variant, which, to our knowledge, has not been previously reported in the literature or included in major genetic databases. Its complete lack of characterization makes it a priority target for future research, as understanding its potential effects on muscle function, metabolism, and performance could reveal novel genotype–phenotype relationships beyond those captured by previously studied *ACTN3* variants.

Beyond these specific polymorphisms, future studies should expand the analysis to additional genes to explore potential interactions with *ACTN3* and their combined influence on performance traits. Increasing both the size and diversity of athletic and clinical cohorts will allow the investigation of role-specific, sex-specific, and clinically relevant differences, while supporting the development of multigenic panels integrating variants potentially relevant to exercise response, performance, and metabolic traits. By combining genetic insights with physiological and clinical data, such panels may contribute to the development of precision sports science and personalized preventive strategies, with the potential to support performance optimization and long-term health across diverse populations. Several limitations should be considered when interpreting the present findings. First, the relatively small sample size and the unequal distribution of genotype groups, particularly for less frequent genotypes, may have limited statistical power and reduced the precision of genotype-specific estimates. Moreover, the number of genotype–phenotype comparisons increased the potential for false-positive findings; therefore, multiple testing correction was applied using the Holm procedure, and only associations that remained significant after correction were considered statistically supported. Second, the presence of different ancestral backgrounds represents a potential source of population stratification. Although ancestry was accounted for in the principal biochemical models, residual confounding cannot be completely excluded, particularly given the observed difference in genotype distribution between ancestry groups. Similarly, the distribution of sex and sport disciplines was not fully balanced across the cohort, which may have introduced additional variability and limits the generalizability of the findings across different athletic populations. Third, the absence of a matched non-athlete control group prevents determining whether the observed genotype and allele distributions are specifically associated with elite athletic status or differ from those expected in the general population. In addition, the cross-sectional design precludes conclusions regarding causality and does not allow assessment of changes in genotype-related phenotypes over time. Another limitation is the potential influence of uncontrolled environmental and physiological factors. Training load, recovery status, nutritional and hormonal status, and other sport-specific factors may affect biochemical markers and physical performance and could not be fully controlled in this retrospective study. Similarly, the exact timing of performance testing and other assessments was not consistently available. Finally, the physical performance dataset was retrospectively obtained from routine assessments conducted over three consecutive competitive seasons. Although all performance assessments were administered by the same strength and conditioning coach, detailed information regarding the uniformity of testing conditions and equipment across seasons was not consistently available. Only one performance record per athlete was available for the present analysis, precluding longitudinal or repeated-measures analyses and limiting the assessment of intra-individual variability over time. Taken together, these limitations warrant a cautious interpretation of the present findings as exploratory. Larger, adequately powered and well-balanced studies, including appropriately matched non-athlete controls and standardized longitudinal assessments, are needed to confirm the observed patterns and clarify the potential contribution of genetic variability to athletic performance and biochemical phenotypes.

## 5. Conclusions

In conclusion, our findings confirm that genetic variability in *ACTN3* can influence key aspects of human physiology. Although not with absolute certainty, in relation to the c.1729C>T (rs1815739), the CC genotype showed a descriptive trend toward higher CK levels and slightly better performance in speed tests, consistent with the previously described association of the C allele with a power-oriented muscle phenotype. However, these associations did not remain statistically significant after correction for multiple comparisons and should therefore be considered exploratory rather than evidence of a confirmed genotype-dependent effect. Conversely, the TT genotype showed numerically lower adiponectin and HDL cholesterol levels, although these differences did not remain statistically significant after correction for multiple comparisons and therefore do not provide sufficient evidence of a less favorable lipid profile or altered metabolic health. Importantly, this study also identified two previously unexplored variants, the synonymous *ACTN3* c.1689A>G (rs2229455) and the novel missense c.1693C>G (p.His565Asp), whose functional and structural effects remain to be elucidated. Future research on these variants could provide deeper insight into their role in muscle function, metabolism, and performance, contributing to a more comprehensive understanding of *ACTN3*-related genotype–phenotype relationships.

## Figures and Tables

**Table 1:** Descriptive genotypic frequency analyses of *ACTN3* c.1729C>T (rs1815739).

Genotype c.1729C>T (rs1815739)	*n* (Athletes)	%
CC (wild-type homozygous)	55	50.46%
CT (heterozygous)	42	38.53%
TT (variant homozygous)	12	11.01%
Total	109	100%

**Table 2:** Descriptive genotypic frequency analyses related to *ACTN3* c.1689A>G (rs2229455).

Genotype c.1689A>G (rs2229455)	*n* (Athletes)	%
AA (wild-type homozygous)	74	67.9%
AG (heterozygous)	30	27.5%
GG (variant homozygous)	5	4.6%
Total	109	100%

**Table 3:** Descriptive genotypic frequency analyses related to *ACTN3* c.1693 C>G (p.His565Asp).

Genotype c.1693C>G (p.His565Asp)	*n* (Athletes)	%
CC (wild-type homozygous)	95	87.2%
CG (heterozygous)	14	12.8%
GG (variant homozygous)	0	0%
Total	109	100%

**Table 4:** Genotype distribution of *ACTN3* polymorphisms (c.1729C>T (rs1815739), c.1689A>G (rs2229455), and c.1693C>G (p.His565Asp)) according to ancestral background in the study population. Data are presented as number of individuals and percentage within each ancestral group (Caucasian and African ancestry). The ancestry-distribution *p*-value was obtained using an exact test; HWE *p*-values were calculated separately within each ancestry group.

Polymorphisms of *ACTN3*	Caucasian (*n* = 75)	African Ancestry (*n* = 34)	Exact *p*; HWE *p* (Caucasian/African Ancestry)
**c.1729C>T (rs1815739)**			**0.049; HWE 0.348/0.052**
CC	32 (42.7%)	23 (67.6%)	
C	34 (45.3%)	8 (23.5%)	
TT	9 (12.0%)	3 (8.8%)	
**c.1689A>G (rs2229455)**			**0.525; HWE 0.444/0.336**
AA	48 (64.0%)	26 (76.5%)	
AG	23 (30.7%)	7 (20.6%)	
GG	4 (5.3%)	1 (2.9%)	
**c.1693C>G (p.His565Asp)**			**0.542; HWE 1.000/1.000**
CC	64 (85.3%)	31 (91.2%)	
CG	11 (14.7%)	3 (8.8%)	

**Table 5:** Associations between the *ACTN3* c.1729C>T (rs1815739) genotype and biochemical and lipid parameters. Normally distributed variables are presented as mean ± SD; non-normally distributed variables are presented as median [IQR]. ε^2^ denotes the Kruskal–Wallis effect size and η^2^ the ANOVA effect size; 95% CIs were obtained by bootstrap resampling. The Holm-adjusted *p*-values apply within the 10-outcome family for this variant. The multivariable *p*-value is Holm-adjusted within the same family for the genotype term in models adjusted for age, BMI, sex, and ancestry.

Outcome	Total (*n* = 109)	CC (*n* = 55)	CT (*n* = 42)	TT (*n* = 12)	Unadjusted *p*	Holm-Adjusted *p*	Effect Size (95% CI)	Multivariable Holm *p*
CK (U/L)	296.0 [182.0–499.0]	417.0 [188.5–615.5]	216.0 [174.0–417.8]	294.3 ± 187.0	0.121	1.000	ε^2^ = 0.021 (0.000–0.128)	1.000
LDH (U/L)	204.0 [180.0–231.0]	208.0 [180.5–239.5]	201.0 [179.2–222.2]	203.5 [183.8–229.5]	0.549	1.000	ε^2^ = 0.000 (0.000–0.080)	1.000
ALP (U/L)	81.0 [71.0–102.0]	85.0 [69.5–105.5]	84.4 ± 20.1	78.5 [70.8–93.2]	0.850	1.000	ε^2^ = 0.000 (0.000–0.061)	1.000
CRP (mg/dL)	0.7 [0.3–1.1]	0.7 [0.3–1.1]	0.5 [0.3–1.0]	1.2 ± 0.8	0.166	1.000	ε^2^ = 0.015 (0.000–0.118)	1.000
NLR (ratio)	1.4 [1.1–1.8]	1.4 [1.1–1.9]	1.4 [1.0–1.7]	1.4 [1.2–1.7]	0.975	1.000	ε^2^ = 0.000 (0.000–0.047)	1.000
Total Cholesterol (mg/dL)	165.9 ± 34.4	165.0 [142.0–187.5]	162.9 ± 34.7	163.0 ± 36.2	0.680	1.000	η^2^ = 0.007 (0.001–0.086)	1.000
LDL Cholesterol (mg/dL)	95.0 [78.0–112.0]	94.0 [80.0–112.0]	98.8 ± 28.6	101.4 ± 30.8	0.944	1.000	ε^2^ = 0.000 (0.000–0.061)	1.000
HDL Cholesterol (mg/dL)	55.0 [49.0–64.0]	58.5 ± 14.1	54.7 ± 9.9	54.2 ± 8.0	0.350	1.000	ε^2^ = 0.001 (0.000–0.096)	1.000
Triglycerides (mg/dL)	63.0 [49.0–75.0]	63.0 [47.5–81.5]	60.6 ± 17.9	63.1 ± 14.7	0.659	1.000	ε^2^ = 0.000 (0.000–0.066)	1.000
Adiponectin (µg/mL)	14.7 ± 2.5	15.1 ± 2.4	14.6 ± 2.4	13.2 ± 2.6	0.050	0.503	η^2^ = 0.055 (0.006–0.188)	0.482

**Table 6:** Associations between the *ACTN3* c.1689A>G (rs2229455) genotype and biochemical and lipid parameters. Normally distributed variables are presented as mean ± SD; non-normally distributed variables are presented as median [IQR]. ε^2^ denotes the Kruskal–Wallis effect size and η^2^ the ANOVA effect size; 95% CIs were obtained by bootstrap resampling. The Holm-adjusted *p*-values apply within the 10-outcome family for this variant. The multivariable *p*-value is Holm-adjusted within the same family for the genotype term in models adjusted for age, BMI, sex, and ancestry.

Outcome	Total (*n* = 109)	AA (*n* = 74)	AG (*n* = 30)	GG (*n* = 5)	Unadjusted *p*	Holm-Adjusted *p*	Effect Size (95% CI)	Multivariable Holm *p*
CK (U/L)	296.0 [182.0–499.0]	301.5 [194.2–548.8]	252.5 [144.2–410.2]	329.4 ± 119.9	0.351	1.000	ε^2^ = 0.001 (0.000–0.086)	1.000
LDH (U/L)	204.0 [180.0–231.0]	205.0 [187.2–235.5]	205.0 [173.5–228.8]	197.2 ± 19.4	0.600	1.000	ε^2^ = 0.000 (0.000–0.062)	1.000
ALP (U/L)	81.0 [71.0–102.0]	80.5 [71.0–102.8]	82.5 [72.0–96.8]	87.0 [73.0–104.0]	0.885	1.000	ε^2^ = 0.000 (0.000–0.058)	1.000
CRP (mg/dL)	0.7 [0.3–1.1]	0.7 [0.3–1.1]	0.7 [0.3–1.1]	0.5 [0.3–0.7]	0.821	1.000	ε^2^ = 0.000 (0.000–0.066)	1.000
NLR (ratio)	1.4 [1.1–1.8]	1.4 [1.1–1.9]	1.4 [0.9–1.7]	1.4 [1.3–1.7]	0.606	1.000	ε^2^ = 0.000 (0.000–0.070)	1.000
Total Cholesterol (mg/dL)	165.9 ± 34.4	163.5 ± 34.5	176.2 ± 33.3	138.6 ± 20.6	0.044	0.355	η^2^ = 0.057 (0.016–0.162)	0.201
LDL Cholesterol (mg/dL)	95.0 [78.0–112.0]	93.5 [78.2–112.0]	104.0 ± 27.7	82.2 ± 19.0	0.134	0.941	ε^2^ = 0.019 (0.000–0.124)	1.000
HDL Cholesterol (mg/dL)	55.0 [49.0–64.0]	55.8 ± 11.3	59.0 [52.2–65.0]	49.0 [37.0–52.0]	0.026	0.237	ε^2^ = 0.050 (0.008–0.145)	0.125
Triglycerides (mg/dL)	63.0 [49.0–75.0]	59.0 [47.2–74.8]	64.9 ± 20.4	77.2 ± 31.2	0.427	1.000	ε^2^ = 0.000 (0.000–0.085)	1.000
Adiponectin (µg/mL)	14.7 ± 2.5	14.9 ± 2.5	14.8 ± 2.4	11.6 ± 2.1	0.015	0.147	η^2^ = 0.076 (0.020–0.169)	0.125

**Table 7:** Associations between the *ACTN3* c.1693C>G (p.His565Asp) genotype and biochemical and lipid parameters. Normally distributed variables are presented as mean ± SD; non-normally distributed variables are presented as median [IQR]. ε^2^ denotes the Kruskal–Wallis effect size and η^2^ the ANOVA effect size; 95% CIs were obtained by bootstrap resampling. The Holm-adjusted *p*-values apply within the 10-outcome family for this variant. The multivariable *p*-value is Holm-adjusted within the same family for the genotype term in models adjusted for age, BMI, sex, and ancestry.

Outcome	Total (*n* = 109)	CC (*n* = 95)	CG (*n* = 14)	Unadjusted *p*	Holm-Adjusted *p*	Effect Size (95% CI)	Multivariable Holm *p*
**CK** (U/L)	296.0 [182.0–499.0]	297.0 [190.0–494.0]	252.5 [109.5–538.8]	0.444	1.000	ε^2^ = 0.000 (0.000–0.075)	1.000
**LDH** (U/L)	204.0 [180.0–231.0]	202.0 [180.5–229.0]	224.0 [180.8–258.0]	0.205	1.000	ε^2^ = 0.006 (0.000–0.093)	0.309
**ALP** (U/L)	81.0 [71.0–102.0]	82.0 [71.0–102.5]	78.5 [72.0–96.5]	0.807	1.000	ε^2^ = 0.000 (0.000–0.034)	1.000
**CRP** (mg/dL)	0.7 [0.3–1.1]	0.6 [0.3–1.1]	0.8 [0.3–2.2]	0.274	1.000	ε^2^ = 0.002 (0.000–0.086)	1.000
**NLR** (ratio)	1.4 [1.1–1.8]	1.4 [1.0–1.8]	1.7 [1.5–2.1]	0.066	0.594	ε^2^ = 0.022 (0.000–0.112)	1.000
**Total Cholesterol** (mg/dL)	165.9 ± 34.4	167.3 ± 35.3	156.1 ± 27.3	0.260	1.000	η^2^ = 0.012 (0.000–0.061)	1.000
**LDL Cholesterol** (mg/dL)	95.0 [78.0–112.0]	95.0 [78.0–116.0]	91.9 ± 22.5	0.452	1.000	ε^2^ = 0.000 (0.000–0.048)	1.000
**HDL Cholesterol** (mg/dL)	55.0 [49.0–64.0]	56.0 [49.0–64.0]	54.0 ± 10.7	0.511	1.000	ε^2^ = 0.000 (0.000–0.049)	1.000
**Triglycerides** (mg/dL)	63.0 [49.0–75.0]	63.0 [49.5–75.0]	62.0 [49.2–72.2]	0.874	1.000	ε^2^ = 0.000 (0.000–0.050)	1.000
**Adiponectin** (µg/mL)	14.7 ± 2.5	14.5 ± 2.5	16.0 ± 2.6	0.044	0.440	η^2^ = 0.037 (0.001–0.150)	1.000

**Table 8:** Associations between the *ACTN3* c.1729C>T (rs1815739) genotype and physical test results. The data are expressed as the means ± SDs.

Physical Tests	Total (*n* = 28)	CC (*n* = 15)	CT (*n* = 10)	TT (*n* = 3)	Unadjusted *p*	Holm-Adjusted *p*
Squat Jump (cm)	60.2 ± 10.1	59.7 ± 11.2	60.6 ± 10.4	61.0 ± 4.7	0.790	1.000
Counter Movement Jump (cm)	61.2 ± 9.4	60.7 ± 10.4	61.5 ± 9.9	62.8 ± 2.7	0.800	1.000
Drop Jump (cm)	56.6 ± 10.1	56.0 ± 11.8	57.1 ± 8.9	58.3 ± 6.0	0.863	1.000
Speed (s × 10m)	1.8 ± 0.1	1.7 ± 0.1	1.9 ± 0.1	1.9 ± 0.0	0.050	0.350
Lateral Bound R (cm)	228.9 ± 15.1	228.2 ± 12.5	224.1 ± 16.6	248.0 ± 8.2	0.053	0.350
Lateral Bound L (cm)	227.9 ± 12.2	229.5 ± 9.1	222.9 ± 16.1	236.3 ± 5.9	0.233	1.000
5-0-5 (s)	2.3 ± 0.1	2.3 ± 0.2	2.3 ± 0.1	2.4 ± 0.1	0.465	1.000

**Table 9:** Associations between the *ACTN3* c.1689A>G (rs2229455) genotype and physical test results. The data are expressed as the means ± SDs.

Physical Tests	Total (*n* = 28)	AA (*n* = 21)	AG (*n* = 6)	GG (*n* = 1)	Unadjusted *p*	Holm-Adjusted *p*
Squat Jump (cm)	60.2 ± 10.1	58.5 ± 8.5	66.5 ± 14.4	57.4	0.500	1.000
Counter Movement Jump (cm)	61.2 ± 9.4	59.5 ± 8.5	67.3 ± 11.6	60.5	0.503	1.000
Drop Jump (cm)	56.6 ± 10.1	54.8 ± 8.5	64.1 ± 13.0	50.0	0.217	1.000
Speed (s × 10m)	1.8 ± 0.1	1.8 ± 0.2	1.7 ± 0.1	1.6	0.081	0.567
Lateral Bound R (cm)	228.9 ± 15.1	228.1 ± 16.3	229.3 ± 11.2	242.0	0.422	1.000
Lateral Bound L (cm)	227.9 ± 12.2	226.6 ± 13.5	231.2 ± 7.0	235.0	0.620	1.000
5-0-5 (s)	2.3 ± 0.1	2.3 ± 0.2	2.2 ± 0.1	2.4	0.250	1.000

**Table 10:** Associations between the *ACTN3* c.1693C>G (p.His565Asp) genotype and physical test results. The data are expressed as the means ±SDs.

Physical Tests	Total (*n* = 28)	CC (*n* = 24)	CG (*n* = 4)	Unadjusted *p*	Holm-Adjusted *p*
Squat Jump (cm)	60.2 ± 10.1	59.8 ± 10.6	62.2 ± 8.0	0.412	1.000
Counter Movement Jump (cm)	61.2 ± 9.4	60.7 ± 10.1	64.5 ± 3.3	0.250	1.000
Drop Jump (cm)	56.6 ± 10.1	56.4 ± 10.6	57.8 ± 7.7	0.555	1.000
Speed (sx10metri)	1.8 ± 0.1	1.8 ± 0.2	1.8 ± 0.1	0.669	1.000
Lateral Bound R (cm)	228.9 ± 15.1	230.5 ± 15.3	219.2 ± 9.9	0.100	0.700
Lateral Bound L (cm)	227.9 ± 12.2	228.4 ± 12.8	225.0 ± 9.3	0.409	1.000
5-0-5 (s)	2.3 ± 0.1	2.3 ± 0.2	2.3 ± 0.2	0.717	1.000

## Data Availability

The original data presented in this study are included in the article.

## Abbreviations

The following abbreviations are used in this manuscript:

*ACTN2*	Alpha-actinin-2
*ACTN3*	Alpha-actinin-3
ALP	Alkaline phosphatase
ANOVA	Analysis of Variance
BMD	Bone Mineral Density
BMI	Body Mass Index
CBC	Complete Blood Count
CK	Creatine Kinase
CRP	C-Reactive Protein
DMD	Duchenne Muscular Dystrophy
DNA	Deoxyribonucleic Acid
EDTA	Ethylenediaminetetraacetic Acid
ELISA	Enzyme-Linked Immunosorbent Assay
GWAS	Genome-Wide Association Studies
HDL	High-Density Lipoprotein
HDL-C	High-Density Lipoprotein Cholesterol
His565Asp	Histidine to Aspartic acid substitution at codon 565
LBM	Lean Body Mass
LDH	Lactate Dehydrogenase
LDL	Low-Density Lipoprotein
LDL-C	Low-Density Lipoprotein Cholesterol
NCBI	National Center for Biotechnology Information
NGS	Next-Generation Sequencing
NLR	Neutrophil-to-Lymphocyte Ratio
PCR	Polymerase Chain Reaction
SD	Standard Deviation
SNP	Single Nucleotide Polymorphism
Thr563=	Synonymous threonine substitution at codon 563
UV	Ultraviolet
